# Fertility Preservation in More than 7000 Male Patients: A Single-Center, Tertiary Care Registry Study over 30 Years

**DOI:** 10.3390/cancers17040689

**Published:** 2025-02-18

**Authors:** Andrea Graziani, Giuseppe Grande, Michel Martin, Donatella Sorio, Federica Finocchi, Sara Corrò, Nicola Passerin, Adriano Presciutti, Antonella Di Mambro, Riccardo Selice, Andrea Garolla, Alberto Ferlin

**Affiliations:** 1 Department of Medicine, University of Padua, 35128 Padova, Italymartin.michel.dr@gmail.com (M.M.); andrea.garolla@unipd.it (A.G.); 2 Unit of Andrology and Reproductive Medicine, Department of Systems Medicine, University Hospital of Padova, 35128 Padova, Italy; giuseppe.grande@aopd.veneto.it (G.G.); donatella.sorio@studenti.unipr.it (D.S.); saracorro@gmail.com (S.C.); nicola.passerin@aopd.veneto.it (N.P.); antonella.dimambro@aopd.veneto.it (A.D.M.); riccardo.selice@aopd.veneto.it (R.S.)

**Keywords:** semen cryopreservation, cancer, male factor infertility, testicular cancer

## Abstract

**Background/Objectives**: Semen cryopreservation has been widely used in recent decades, mainly in patients with male factor infertility (MFI) and patients with cancer (to preserve their fertility potential before undergoing gonadotoxic treatments, such as chemotherapy or radiotherapy). **Methods**: we evaluated the temporal trend and the existence of factors determining the usage of cryopreserved semen (in order, for instance, to conceive using assisted reproduction techniques (ARTs)). We evaluated 7044 patients who cryopreserved since 1991, evaluating clinical information such as the diagnosis, age, the method with which the collection took place and the number of pick-ups for use in ART. **Results**: The mean age of the patients was 33.12 ± 8.41 years. The most common cancers were testicular (1758/3262) and hematological cancers (942/3262). Patients who underwent cryopreservation for MFI picked up their samples much more than neoplastic patients (42.15% vs. 8.55%). Patients 35–40 years old picked up their samples more frequently when compared with other age groups. Moreover, we found several data regarding the sub-types of cancers and the methods of semen collection. **Conclusions**: According to our results, the indication for semen cryopreservation, age and the method of collection might represent a simple way to predict the future use of semen for ART.

## Simple Summary

Semen cryopreservation has been widely used in recent decades, mainly in patients with male factor infertility (MFI) and patients with cancer (to preserve their fertility potential before undergoing gonadotoxic treatments, such as chemotherapy or radiotherapy). We evaluated the temporal trend and the existence of factors determining the usage of cryopreserved semen in a population of 7044 patients who cryopreserved since 1991, evaluating clinical information such as the diagnosis, age, the method with which the collection took place and the number of pick-ups for use in ART. The most common cancers were testicular and hematological cancers. Patients who underwent cryopreservation for MFI picked up their samples much more than neoplastic patients. Patients 35–40 years old picked up their samples more frequently when compared with other age groups. According to our results, the indication for semen cryopreservation, age and the method of collection might represent a simple way to predict the future use of semen for ART.

## 1. Introduction

Semen cryopreservation is a technique which allows male gametes to be frozen and maintained in liquid nitrogen, even long-term, preserving/enhancing the chance of future conception [1]. It is proposed mainly for patients who have to undergo genotoxic therapy (radiotherapy or chemotherapy) or surgical interventions which could irreversibly compromise fertility and for patients with male factor infertility (MFI)—in particular, patients with cryptozoospermia, severe oligozoospermia or azoospermia [2,3]. Moreover, it can be used in patients on active duty in a dangerous occupation for male fertility, patients with ejaculatory or sexual disorders and transgender assigned male at birth (AMAB) subjects before undergoing gender-affirming hormonal therapy [4,5].

As said above, two main reasons for semen cryopreservation are MFI and cancer. Regarding MFI, it should be underlined that patients should be counselled on cryopreservation after a proper, meticulous and complete diagnostic—and even therapeutic—program in order to evaluate and possibly find any treatable cause of MFI and lower the extent of the so-called “idiopathic” MFI [6,7,8].

Regarding cancer, cryopreservation of semen is pivotal for fertility preservation for young adults and adults with cancer [9]. Most patients who cryopreserve are affected by testicular cancer or hematological malignancies. Regarding testicular cancer, it has to be considered that it is the most common cancer in young adults [10] with a high overall 5-year survival rate—above 95% [11].

The number of frozen devices (or paillettes), which contain the cryopreserved semen, depends on the quality of the sample with regard to maximizing the chances of achieving a pregnancy after assisted reproductive techniques (ARTs) [12].

The first rudimentary attempt at semen cryopreservation dates back to over two centuries ago when the Italian Spallanzani attempted to conserve spermatozoa by cooling them in the snow [13]. Over the following decades, several attempts followed, but they did not lead to significant success until 1949 when the cryoprotective function of glycerol was discovered. This allowed a wide diffusion of semen cryopreservation.

Given the intrinsic complexity of the cryopreservation process, it should be carried out in reference centers by physicians and biologists trained in the clinical and laboratory aspects of the procedure, respectively.

The objective of our study was to evaluate the temporal trend regarding male fertility preservation and the existence of factors determining the use of cryopreserved gametes (semen) in the Unit of Andrology and Reproductive Medicine, University Hospital of Padua, Italy.

## 2. Materials and Methods

A single-center, retrospective registry study was performed at the Unit of Andrology and Reproductive Medicine, Department of Medicine, University Hospital of Padua, Italy. Patients who cryopreserve semen in our center are annually followed up to ascertain their desire to maintain the cryopreserved material, to determine their desire to use their semen for assisted reproductive techniques (ARTs) and to evaluate hormonal and testicular parameters. This aspect is particularly important in patients with cancer who might develop hormonal or testicular negative consequences after the gonadotoxic therapy. Furthermore, during follow-up, it is possible to evaluate the change in semen parameters following gonadotoxic therapies and, in most cases, observe a full or partial recovery of testicular function, further allowing the possibility of natural fertility (avoiding ART).

Starting from these premises, we retrospectively evaluated patients who cryopreserved male gametes (sperm cells) between November 1991 and November 2023. For each of these patients, we retrospectively collected information regarding the diagnosis (the reason for semen cryopreservation—MFI, cancer or other reason), age at the time of cryopreservation, the method through which the semen collection was carried out (classical seminal fluid cryopreservation, testicular fine needle aspiration, testicular biopsy or semen collection from urine), the number of semen pick-ups for use in ART and the number patients who have requested to withdraw from the maintenance of cryopreserved samples, thus requiring the elimination of semen samples from our bank. Testicular fine needle aspiration and/or testicular biopsy were performed in azoospermic MFI patients, while collection from urine was performed in patients with retrograde ejaculation.

The study protocol followed the standard clinical approach and the principles outlined in the Declaration of Helsinki. Informed consent to collect the data anonymously for scientific purposes was obtained from the study participants. IRB exemption for registry studies was requested according to COPE recommendations.

Statistical Package for the Social Sciences software (IBM SPSS Statistics, Version 29.0, Armonk, NY, USA) was used for statistical analysis. Before statistical analysis, the Kolmogorov–Smirnov test was performed in order to evaluate the normality of the distribution of continuous variables. As part of the statistical analysis conducted on the categorical variables of this study, we used the chi-square test to evaluate the disparities between observed and expected frequency distributions. The difference between continuous variables was assessed using Student’s *t* tests and ANOVA. A *p* value < 0.05 was considered statistically significant.

## 3. Results

We obtained data regarding 7044 patients, all patients who cryopreserved their semen between November 1991 and November 2023 in our unit.

We divided patients into three categories, according to the reason for semen cryopreservation: patients with MFI, patients with a new diagnosis of cancer and patients who preserved their fertility for other reasons (i.e., patients with retrograde ejaculation and patients undergoing non-oncological gonadotoxic therapies—i.e., for rheumatological reasons and AMAB subjects).

**Figure 1:**
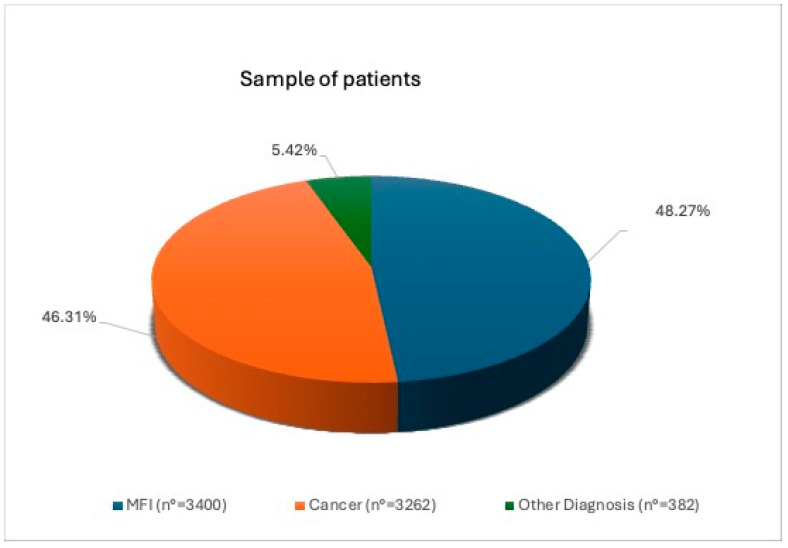
Overall representation of the sample of patients who cryopreserved semen in our unit. Abbreviations: MFI: male factor infertility.

Patients with MFI were 3400/7044 (48.27%), patients with cancer were 3262/7044 (46.31%) and patients who preserved their fertility for other causes were 382/7044 (5.42%) of the total (Figure 1).

**Figure 2:**
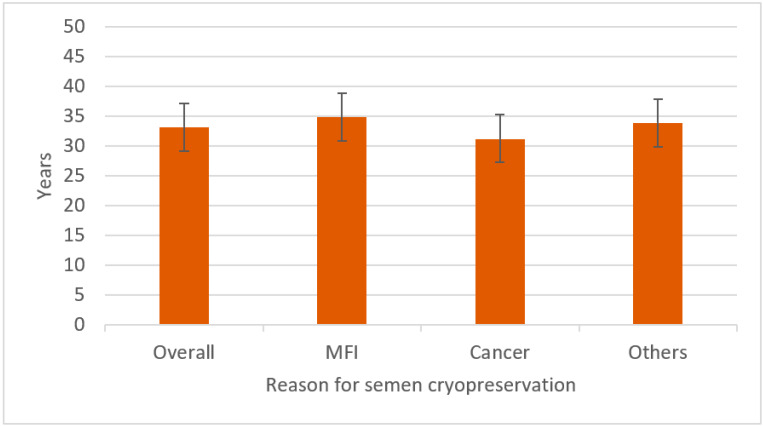
Age of the population at the time of semen cryopreservation, considering the overall.

The mean age of the patients at the time of the cryopreservation was 33.12 ± 8.41 years. Considering the overall population, the age group between 30 and 34 years, representing 24.56% of the study population (1730/7044), was the largest. The population was therefore stratified based on the age at the time of the same (Figure 2). Patients with MFI were older than those in the other two categories of patients at the time of the cryopreservation (*p* < 0.001), while patients with a new diagnosis of cancer were the youngest among the three groups (*p* < 0.001).

**Figure 3:**
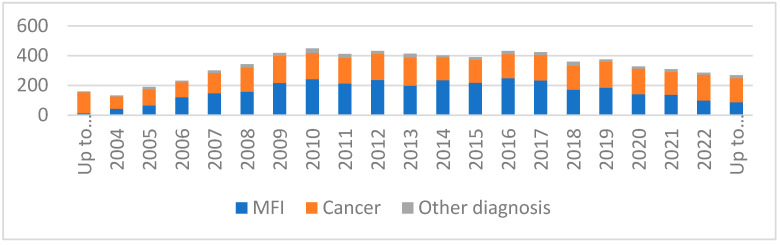
Overall representation of patients who cryopreserved semen in our unit over the years.

Regarding the temporal trend in semen cryopreservation, the number of cryopreservation procedures performed has changed significantly over the years. In particular, between 2004 and 2010, the number of cryopreservation procedures gradually increased, remaining essentially stable between 2012 and 2019 and then recording a progressive decline from 2020 to today. The highest number of cryopreservation procedures occurred in 2012, in which 442 new patients were recorded. The number of new cryopreservation procedures in patients with cancer remained essentially stable over the years; on the contrary, there was a decrease in cryopreservation procedures in patients with MFI (in particular, from 2020 up to November 2023) (Figure 3).

The cryopreserved material was also divided based on the method of collection (Table 1). As previously shown, four different methods were used for the recovery of gametes: seminal fluid, testicular fine needle aspiration, testicular biopsy and semen collection from urine. Among patients with cancer, 98.5% (3213/3262) of cryopreservation procedures consisted of the collection of gametes from seminal fluid, via biopsy in 1.1% (36/3262), via needle aspiration in 0.31% (10/3262) and from the patient’s urine in 0.09% (3/3262) of cases. Cryopreserved gametes from patients with MFI were collected through the seminal fluid in 61.15% (2079/3400), through testicular biopsy in 29.94% (1018/3400), through testicular fine needle aspiration in 8.47% (288/3400) and through recovery from urine in 0.44% (15/3400) of cases. Finally, considering patients with other diseases, 94.24% (360/382) of the cryopreservation procedures involved the collection of seminal fluid, 2.09% (8/382) testicular biopsy, 3.4% (13/382) testicular fine needle aspiration and 0.26% (1/382) collection from urine.

The number of pick-ups per year in the time period analyzed in this study was variable. The highest number of withdrawals occurred in 2011, with 138 patients who picked up their cryopreserved semen for the first time.

**Figure 4:**
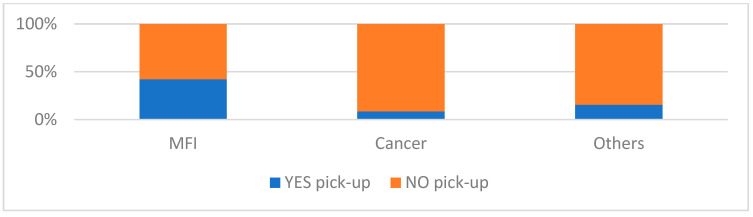
The number of pick-ups (expressed as total percentages) analyzed by observing the population according to the indication for cryopreservation.

The number of pick-ups was therefore analyzed by observing the population according to the indication for cryopreservation and the age at the time of the cryopreservation (Figure 4). Patients with MFI are characterized by a significantly greater number of pick-ups (42.15%, 1433/3400) than patients with a diagnosis of cancer (8.55%, 279/3262) (*p* < 0.05), while patients with other diagnoses were instead characterized by an intermediate pick-up rate (15.45%, 59/382).

Consequently, we obtained the pick-up rates of each subcategory of patients diagnosed with cancer. Pick-up rates were 7.91% in patients with testicular cancer, 7.96% in patients with hematological cancers and 11.57% in patients with other types of cancer (*p* < 0.05).

Regarding the association between age and pick-ups, by dividing the population into different age subgroups, it was observed that patients belonging to younger age groups at the time of cryopreservation had a lower pick-up rate (and therefore a lower use) of cryopreserved semen compared to intermediate-age patients and older patients, with a significant difference (*p* < 0.05). The subgroup of patients aged between 35 and 40 years had the highest pick-up rate (36.05%), while patients under 20 had the lowest rate (1.98%). Considering both the reason for cryopreservation and age, the highest pick-up rate was found in patients with MFI aged 30–34 years (49.77%), as reported in Table 2.

**Figure 5:**
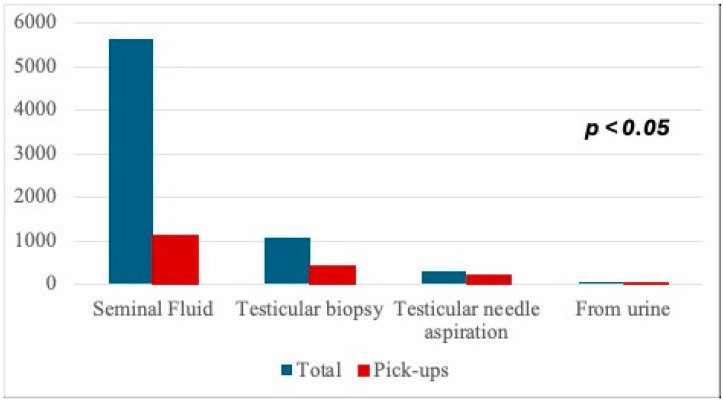
Association between method of semen collection for cryopreservation and rates of pick-up.

Considering the cryopreservation method, we found that patients who underwent sperm cell collection for preservation by testicular fine needle aspiration and the recovery of male gametes from urine, 73.68% (14/19) and 68.17% (212/311), respectively, more frequently used their samples, while gametes obtained by testicular biopsy or from traditional seminal fluid were picked up in 38.42% (408/1062) and in 20.12% (1137/5652) of cases (Figure 5), respectively.

This general trend has been confirmed in the population of patients with MFI. In detail, in this subgroup of patients, as previously shown, 1433/3400 patients (42.15%) picked up their semen. Out of these 1433 patients, 830/1433 underwent collection from seminal fluid, 388/1433 by testicular biopsy, 203/1433 by testicular fine needle aspiration and 12/1433 from urine. Therefore, considering only patients with MFI and the method of collection (shown in Table 1), we found that infertile patients who cryopreserved via testicular biopsy picked up in 38.1% of cases, from seminal fluid in 39.9% of cases, by testicular needle aspiration in 70.5% of cases and from urine in 80.0% of cases, with the highest rate of pick-up in patients undergoing testicular aspiration and collection from urine.

Furthermore, we considered the duration of cryopreservation according to the diagnosis, the age of patients and the method of collection.

**Figure 6:**
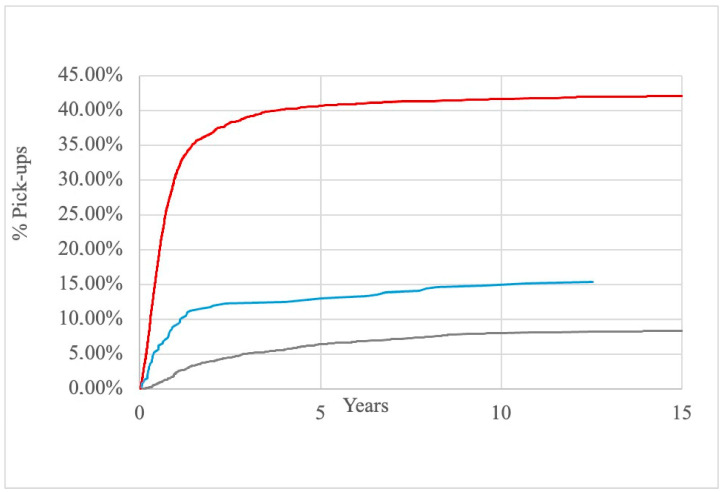
Association between the time of the pick-up of cryopreserved material and the reason for cryopreservation. Legend: red line: patients with MFI; blue line: patients with other diagnoses; gray line: patients with cancer.

When considering the diagnosis, we found a significant difference (*p* < 0.05) in the length of cryopreservation (Figure 6). Patients with MFI waited a shorter interval of time before picking up cryopreserved samples (1.126 ± 1.88 years) compared to patients with other diagnoses (2.086 ± 3.74 years). Patients with cancer, on the contrary, maintained the samples in the cell bank for a longer interval of time (3.64 ± 3.01 years).

Moreover, when considering the age of patients, we observed that the category of patients aged between 45 and 49 was characterized by the shortest average interval (0.85 ± 0.97 years); on the contrary, the group aged below 20 was found to be the one with the longest interval (11.89 ± 7.05 years).

Finally, when considering the method of semen collection, it was found that semen obtained through seminal fluid was picked up after an average of 1.89 ± 2.83 years, gametes collected by testicular biopsy were picked up after 0.98 ± 1.60 years, gametes collected by testicular fine needle aspiration after 0.91 ± 1.35 years and spermatozoa cryopreserved after being obtained from urine after 1.35 ± 2.45 years.

Therefore, according to our overall data, older patients tend to pick up more and after a shorter interval than patients in the younger subgroups (*p* < 0.001). This is particularly true when comparing older patients with MFI and younger patients with cancer (Table 3).

**Figure 7:**
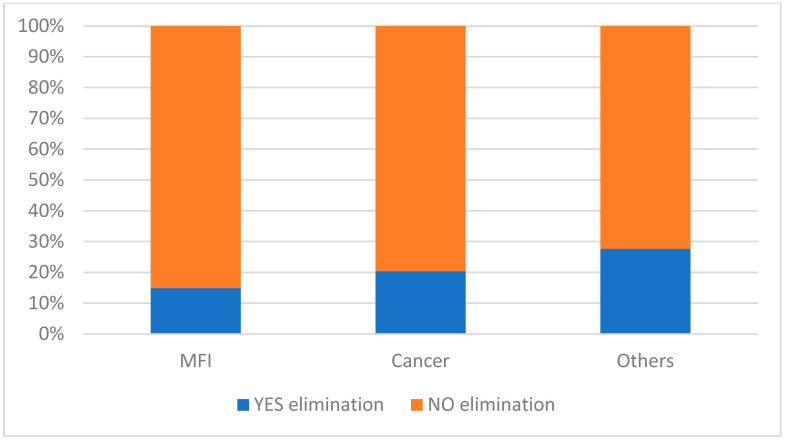
Rate of sample elimination according to the reason for semen cryopreservation.

We finally evaluated the number of eliminations of semen samples from our bank due to patients’ will (mainly because they were not interested in fertility anymore or their natural fertility had been restored). A total of 18.1% of patients decided to eliminate their cryopreserved semen (1277/7044). According to the age of patients, the subclasses of patients who had the highest elimination rates were 30–35 years (20.9%), 35–40 years (21.8%) and 40–45 years (23.1%), while the lowest elimination rate was found in patients under 20 years (8.5%). According to the diagnosis, 20.4% of patients who cryopreserved with cancer (665/3262) and 14.9% of patients who cryopreserved due to MFI (506/3400) decided to eliminate their samples (*p* < 0.05) (Figure 7). Finally, according to the method of collection, we found that the percentage of patients who decided to eliminate their samples was 20.4% (1155/5652) for patients who underwent sample collection by masturbation, 17.7% (55/311) for patients who underwent sperm cell collection by testicular fine needle aspiration, 15.8% (3/19) for patients who underwent sperm cell collection from urine and 6.0% (64/1062) for patients who underwent sperm cell collection by testicular biopsy.

## 4. Discussion

Semen cryopreservation has been widely used, especially in recent decades, to preserve male fertility, mainly in patients with MFI and in patients with cancer (in the latter category, in order to preserve fertility potential before undergoing gonadotoxic treatments, such as chemotherapy or radiotherapy).

In our study, we evaluated the temporal trend and the existence of factors determining the usage of cryopreserved semen (i.e., age at the time of semen cryopreservation, method of semen collection, indication to semen cryopreservation…).

In our cohort of patients, we found that the most represented category is that of patients with MFI (3400/7044, 48.27%), followed by patients with cancer (3262/7044, 46,31%) and patients with other conditions requiring fertility preservation (382/7044, 5.42%). Regarding patients with cancer, the most represented subgroup is that of patients diagnosed with testicular cancer. The mean age of the patients at the time of the cryopreservation was 3.12 ± 8.41 years, with the age group between 30 and 34 years being the most represented. A very recent study evaluating 2732 oncology patients who cryopreserved their semen (mean age 29.8 years) found—in line with our results—that the most common diagnosis among them was testicular cancer (29.5%) [14].

Patients with MFI were found to be older than those in the other two categories of patients at the time of the cryopreservation, while patients with a new diagnosis of cancer were the youngest among the three groups.

Regarding the temporal trend in semen cryopreservation, we recorded a progressive decline from 2020 to today, in particular, in patients with MFI. We have ascribed this trend to the SARS-Cov-2 pandemic infection. On the other hand, the number of semen cryopreservation procedures in patients with cancer was overall stable and did not show a trend of reduction.

Furthermore, we analyzed the impact of age, diagnosis and method of collection on the usage of cryopreservation. Patients with MFI are characterized by a greater number of pick-ups (42.15%, 1433/3400) than patients with a diagnosis of cancer (8.55%, 279/3262). In a previous study evaluating 1524 men, Ferrari et al. reported that only 9.4% of cancer survivors who had previously cryopreserved their semen picked up sperm for ART [15]. Moreover, Dearing et al. reported a 6% sperm pick-up rate among 3062 oncology patients who stored samples from 1976 to 2009 [16]. A more recent study reported an overall sperm usage among cancer survivors of 19.3% [3]. A recent meta-analysis [17], dealing with fertility preservation in adult male patients with cancer, included 69 non-randomized studies and found an observed utilization rate of frozen sperm of 9%.

When considering the association between age and pick-up rate, we found that patients belonging to younger age groups at the time of cryopreservation had a lower pick-up rate (and therefore a lower use) of cryopreserved semen compared to intermediate-age patients and older patients, with the subgroup of patients aged between 35 and 40 years having the highest pick-up rate.

A previous study by Bitan et al. [3], evaluating 1490 men who cryopreserved semen for three decades, reported that increasing age was associated with progressive increase in sperm usage rate (increase in pick-up rate) and shorter preservation period. Another study evaluated 279 testicular cancer survivors and reported that only one-third of them underwent sperm cryopreservation before oncological treatment, with 11% of them using sperm for ART, namely, younger patients and those with a lower body mass index at the time of semen cryopreservation [18].

Finally, when evaluating the method of collection, we found that the use of more “invasive” methods, such as testicular biopsy and testicular needle aspiration, were associated with higher pick-up rates, mainly in patients with MFI. In fact, these procedures are usually proposed for semen cryopreservation in patients who are actively trying to conceive and desire to rapidly proceed with ART. Since they are invasive procedures, it is expected that they are undertaken by patients undergoing ART so that collected sperm samples collected are picked up and therefore used with a high frequency. It has to be remembered that these procedures, especially testicular fine needle aspiration cytology, besides their “therapeutic role” for sperm usage in ART, are currently used as diagnostic and diagnostic tools in the field of MFI [6,19].

Similar considerations may be postulated for the high rate of use reported for samples collected from urine; this is a procedure generally undertaken by patients with retrograde ejaculation currently undergoing ART cycles or with prospective of ART cycles.

Finally, we evaluated the duration of cryopreservation according to the diagnosis, the age of patients and the method of collection. Patients with MFI waited a shorter interval of time before picking up their cryopreserved samples (1.126 ± 1.88 years) compared to patients with other diagnoses (2.086 ± 3.74 years). Regarding patients with cancer, they waited a longer interval of time (3.64 ± 3.01 years). When considering the age of patients, we observed that the category of patients aged between 45 and 49 was characterized by the shortest average interval; on the contrary, the group aged below 20 was found to be the one with the longest interval. When considering the method of semen collection, it was found that semen obtained through seminal fluid was picked up after a longer interval of time than semen obtained by the other method of collection, with the two “invasive” methods being associated with shorter intervals of time.

Therefore, according to our overall data, older patients tend to pick up more and after a shorter interval than patients in the younger subgroups (*p* < 0.001). This is particularly true when considering older patients with MFI and younger patients with cancer.

Cancer therapy or the cancer itself might cause problems with male fertility for affected patients, and MFI is a risk associated with several treatment regimens. Despite ongoing research in cancer treatment therapies and the evolution of treatment regimens to be less gonadotoxic, it is still difficult to predict whether a patient is at risk of permanent sterility or how long it will take for their sperm production to resume [20].

Cryopreservation of sperm and/or testicular tissue is standard care for fertility preservation that has to be discussed with young adults and adults with cancer [21]. Therefore, patient counseling prior to cancer treatment by an oncologist and/or fertility specialist is crucial [22]. The long-term viability of cryopreserved semen is of paramount importance for younger men, who often undergo sperm cryopreservation for gonadotoxic treatments, as there might be many years between the time when the semen is frozen and when it is used in ART [23].

Indeed, as previously said, proper clinical practice requires, after a period, a re-evaluation of the overall fertility status of men in order to evaluate whether a situation of normal fertility has been restored, allowing a natural pregnancy to be achieved and thus elimination of the cryopreserved semen. This is suggested even by guidelines dealing with this topic [21].

In fact, the effects of gonadotoxic treatments depend on a variety of factors, including the patient age, type, cumulative dosage of chemotherapeutic agents, site and dosage of irradiation, the potential synergic interaction of radio-/chemotherapy, site and type of surgery as well as treatment duration and the presence of previous underlying testicular diseases [21], with all these factors determining the stages of spermatogenesis suppressed and the duration of suppression.

According to our data, about one-fourth to one-fifth of patients aged 30–45 decided to eliminate their semen samples.

Although we did not collect semen analysis performed during follow-up in this registry study, these data together seem to suggest that several patients with cancer (mean age at diagnosis: 31.24 ± 8.52 years) may, in the following years, return to their normal fertility and therefore decide to eliminate cryopreserved semen. We therefore suggest performing a complete fertility evaluation (hormones, semen analysis, testis ultrasound, sperm DNA aneuploidy evaluation and sperm DNA fragmentation evaluation) about two years after the final oncological procedure (i.e., two years after the end of chemotherapy/radiotherapy). Nevertheless, the timeline depends on the type of cancer and cancer treatment combination used. As a result, it is very difficult to ascribe a specific time to recovery of “normal sperm”, with many authors recommending 18 to 24 months of continued contraception before couples either resume unprotected intercourse or attempt to conceive spontaneously [21].

The limitations of our study include the retrospective nature of the evaluation, the lack of data regarding semen cryopreservation in pre-pubertal patients (in contrast to other studies, such as [24,25]), the lack of data regarding hormonal and semen parameters at the first evaluation and during follow-up and the lack of data regarding pregnancies in patients who picked up semen for ART.

## 5. Conclusions

Sperm cryopreservation is pivotal for fertility preservation in young adults/adults with cancer and in patients with severe forms of MFI. In our study, we found that patients with MFI, with respect to patients with cancer, have higher pick-up rates of cryopreserved semen and lower interval of time between cryopreservation and sperm usage. Moreover, we found that age is a parameter which influences the pick-up rate, with younger age being associated with a lower pick-up rate. Finally, more invasive methods of semen collection were found to be associated with a lower interval of time before pick-up for usage in ART.

## Figures and Tables

**Table 1:** Method of collection of cryopreserved semen according to the diagnosis.

Method of Semen Collection	Patients (Total)	Patients with Cancer	Patients with MFI	Patients with Other Diagnoses
Semen fluid	80.24% (5652/7044)	98.5% (3213/3262)	61.15% (2079/3400)	94.24% (360/382)
Testicular biopsy	15.08% (1062/7044)	1.1% (36/3262)	29.94% (1018/3400)	2.09% (8/382)
Testicular cytology	4.42% (311/7044)	0.31% (10/3262)	8.47% (288/3400)	3.4% (13/382)
Collection of semen from urine	0.27% (19/7044)	0.09% (3/3262)	0.44% (15/3400)	0.26% (1/382)

**Table 2:** Association between age at the time of cryopreservation, overall pick-up rate and the reason for seme cryopreservation.

Age (Years)	Pick-Up Rate	Cancer	MFI	Other Diagnosis
<20	1.98% (10/504)	1.97% (6/305)	2.34% (4/171)	0% (0/28)
20–24	4.92% (39/753)	3.82% (19/498)	7.42% (19/256)	2.56 (1/39)
25–29	16.08% (172/1070)	6.36% (42/660)	35.21% (125/355)	9.09% (5/55)
30–34	30.17% (522/1730)	9.28% (72/776)	49.77% (433/870)	20.24% (17/84)
35–39	36.05% (558/1548)	14.73% (80/543)	49.62% (461/929)	22.37 (17/76)
40–44	33.30% (293/880)	10.32% (29/281)	47.72% (252/528)	18.31% (13/71)
45–49	35.28% (121/343)	19.83% (24/121)	45.59% (93/204)	22.22% (4/18)
>50	31.25% (55/176)	8.97% (7/78)	52.87% (46/87)	18.18% (2/11)

**Table 3:** Intervals of pick-ups (years) according to the age of patients and the diagnosis.

Age (Years)	Cancer (Years)	MFI (Years)	Other Diagnosis (Years)
<20	12.92 ± 8.29 (n = 6)	10.19 ± 5.32 (n = 4)	(n = 0)
20–25	7.29 ± 4.08 (n = 19)	6.39 ± 5.18 (n = 19)	7.92 (n = 1)
25–30	5.62 ± 3.64 (n = 42)	2.49 ± 3.40 (n = 125)	4.14 ± 4.95 (n = 5)
30–35	3.49 ± 3.26 (n = 72)	1.04 ± 1.32 (n = 433)	1.71 ± 2.38 (n = 17)
35–40	2.65 ± 2.75 (n = 80)	0.86 ± 1.34 (n = 461)	1.48 ± 1.98 (n = 17)
40–45	1.67 ± 1.49 (n = 29)	0.75 ± 0.89 (n = 252)	2.03 ± 2.64 (n = 13)
45–50	1.66 ± 1.15 (n = 24)	0.66 ± 0.83 (n = 93)	0.26 ± 0.17 (n = 4)
>50	1.75 ± 1.33 yrs (n = 7)	0.75 ± 0.82 yrs (n = 46)	6.37 ± 8.70 yrs (n = 2)

## Data Availability

The original contributions presented in this study are included in the article. Further inquiries can be directed to the corresponding author.
